# Detection of Islet Cell Immune Reactivity with Low Glycemic Index Foods: Is This a Concern for Type 1 Diabetes?

**DOI:** 10.1155/2017/4124967

**Published:** 2017-07-27

**Authors:** Datis Kharrazian, Martha Herbert, Aristo Vojdani

**Affiliations:** ^1^Harvard Medical School, Boston, MA, USA; ^2^TRANSCEND Research, Department of Neurology, Massachusetts General Hospital, Charlestown, Boston, MA 02129, USA; ^3^Department of Preventive Medicine, Loma Linda University School of Medicine, Loma Linda, CA, USA; ^4^Immunosciences Laboratory, Inc., Los Angeles, CA, USA

## Abstract

Dietary management of autoimmune diabetes includes low glycemic foods classified from the glycemic index, but it does not consider the role that immunoreactive foods may play with the immunological etiology of the disease. We measured the reactivity of either monoclonal or polyclonal affinity-purified antibodies to insulin, insulin receptor alpha, insulin receptor beta, zinc transporter 8 (ZnT8), tyrosine phosphatase-based islet antigen 2 (IA2), and glutamic acid decarboxylase (GAD) 65 and 67 against 204 dietary proteins that are commonly consumed. Dietary protein determinants included unmodified (raw) and modified (cooked and roasted) foods, herbs, spices, food gums, brewed beverages, and additives. There was no immune reactivity between insulin or insulin receptor beta and dietary proteins. However, we identified strong to moderate immunological reactivity with antibodies against insulin receptor alpha, ZnT8, IA2, GAD-65, and GAD-67 with several dietary proteins. We also identified 49 dietary proteins found in foods classified as low glycemic foods with immune reactivity to autoimmune target sites. Laboratory analysis of immunological cross-reactivity between pancreas target sites and dietary proteins is the initial step necessary in determining whether dietary proteins may play a potential immunoreactive role in autoimmune diabetes.

## 1. Introduction

Diabetes mellitus, commonly called diabetes, is a group of metabolic diseases in which the body experiences unusually high blood sugar levels over an extended period of time. Diabetes is a leading cause of death and disability in the United States and affects more than 9.3% of the population [1]. The total cost of diabetes in the United States is estimated at more than $245 billion annually [2]. There are four broad recognized categories of diabetes:

- Type 1 diabetes (T1D) is a result of the pancreas' failure to produce sufficient insulin. The pancreas is a glandular organ that not only secretes digestive enzymes but also produces important hormones. These hormones are produced inside the pancreas by clumps of cells called islet cells. Of the five kinds of islet cells, alpha, beta, delta, gamma (PP), and epsilon, only beta islet cells produce the hormone insulin, which regulates blood sugar levels. In an autoimmune condition, the body's own immune system can mistakenly attack and damage or destroy beta islet cells, leading to a reduction of the insulin needed to regulate the body's blood sugar levels [3].
- Type 2 diabetes is a condition in which the cells of the body fail to respond to insulin properly, usually caused by excessive body weight and lack of exercise. Lack of insulin may also develop as the disease progresses [3, 4].
- Gestational diabetes occurs when pregnant women without a previous history of diabetes develop high blood sugar levels [3].
- Other specific types are a collection of a few dozen different causes [5].

Many new cases of diabetes are due to an overlooked autoimmune etiology called latent autoimmune diabetes of adulthood (LADA), which is often misdiagnosed as type 2 diabetes [6]. In the early stages of LADA, progressive autoimmune destruction of islet cells leads to hyperglycemia that does not yet require insulin. As a result, it is commonly misdiagnosed as type 2 diabetes [7]. LADA accounts for 10% of all cases of diabetes and 50% of nonobese diabetes [8]. Despite the autoimmune pathophysiology of LADA, current medication and treatment approaches are focused solely on hyperglycemia control rather than clinical strategies to prevent progression of autoimmune destruction of islet cells [9]. Progression of islet cell destruction from autoimmunity leads to insulin therapy and associated complications of autoimmunity [10]. Experts have expressed the need to identify new therapeutic applications for individuals who are diagnosed with LADA, as the glucose-control model is not sufficiently effective with these patients [9]. The standard practice of using the glycemic index restriction diet for autoimmune diabetes does not consider dietary proteins that have the potential to immunologically cross-react with pancreatic islet cells.

LADA and juvenile type 1 diabetes are characterized by autoimmune destruction hyperglycemia. The pathogenic model in which antigens initiate and drive the process is currently under investigation [11, 12]. Other pathogenic organisms, such as rubella infection, enteroviruses, human cytomegalovirus, and rotavirus, have all been suggested to play a role in cross-reactivity leading to pancreatic islet cell destruction [13]. Cross-reactivity is thought to occur when antigens share amino acid sequence homology with self-tissue proteins in susceptible hosts and has been theorized as a trigger for tissue-specific autoimmune diseases [14–16]. Immunological cross-reactivity was first identified in 1942 when it was found that individuals sensitized to pollen allergens developed immune reactivity to specific fruits [17]. Further study found that cross-reactivity with pollen could also occur to human tissue target proteins [18].

Dietary protein cross-reactivity research has received limited attention in type 1 diabetes. Current research has mostly been limited to gluten and milk proteins as potential sources of pancreatic islet cell destruction. Cow's milk albumin has also been suggested in the etiology of type 1 diabetes due to milk peptide antibody (bovine serum albumin antibodies) binding to beta-cell-specific surface protein and promoting islet cell destruction [19–21]. Additionally, the prevalence of celiac disease in adult type 1 diabetic patients was found to be approximately 10.5% [22]. In one study, antibodies to the wheat storage globulin Glb1 were found in the serum of diabetic patients, but not in age-, sex-, nor HLA-DQ-matched controls. This provides a first candidate wheat protein that is not only antigenic in diabetic rats and human patients but also closely linked to autoimmune attack on the pancreas [23]. A review paper found that studies in animal models and infant exposure to gluten demonstrated diabetogenic potential of gluten exposure. Some studies now suggest a gluten-free diet that may preserve beta cell function [24].

It is possible that other food proteins play a role in pancreatic cell cross-reactivity leading to islet cell destruction. In this study, we evaluated the potential for dietary protein cross-reactivity with insulin and pancreatic target sites by evaluating immune reactivity between target-specific antibodies and purified dietary proteins shown in Table 1. We measured immune reactivity of either target-specific monoclonal or polyclonal antibodies for insulin, insulin receptor alpha (IR-A), insulin receptor beta (IR-B), zinc transporter 8 (ZnT8), islet antigen 2 (IA2), and glutamic acid decarboxylase (GAD) 65 and 67 antibodies against 204 food items that are commonly consumed. Insulin is a signaling hormone produced by the beta cells of the pancreatic islets that allows for the utilization of glucose across cell membranes for bioenergetics metabolism. Some dietary food proteins have been shown to promote autoimmunity to insulin via cross-reactivity [25]. Insulin receptors are tyrosine kinase receptors that play a key role in glucose homeostasis. There are two isomers of insulin receptors, alpha (IR-A) and beta (IR-B) [26, 27]. Antibodies reactive with islet cells have long been associated with T1D and are considered early markers for the disease [28]. Islet antigen 2 (IA2), formerly known as islet cell antigen 512 (ICA512), is a common tyrosine phosphatase-related autoantigen located in the insulin secretory granule membrane in beta cells [29]. Zinc transporter 8 (ZnT8) is the most consistent zinc transporter expressed by beta cells [29]. It has been found as an autoantigen in a high percentage of new-onset T1D patients [30]. Autoantibodies and T-cell responses against ZnT8 are produced in patients that develop autoimmune diabetes [29]. Glutamic acid decarboxylase (GAD) exists in two isoforms (GAD-65 and 67) and both are found in pancreatic tissue and support neuroendocrine modulation of insulin release [31–33].

The determination of food proteins that cross-react with islet cell antigens may identify dietary proteins that are potential triggers for a subset of individuals with autoimmune diabetes.

## 2. Materials and Methods

### 2.1. Polyclonal and Monoclonal Antibodies

Mouse monoclonal anti-insulin antibodies, affinity-purified rabbit polyclonal insulin receptor alpha (IR-A) antibodies, mouse monoclonal insulin receptor beta (IR-B) antibodies, affinity-purified rabbit polyclonal GAD-65 antibodies, and affinity-purified rabbit polyclonal GAD-67 antibodies were purchased from Abcam (Cambridge, MA, USA). Mouse monoclonal ZnT8 antibodies were purchased from Santa Cruz Biotechnology, Inc. (Dallas, TX, USA), and mouse monoclonal IA2 antibodies were purchased from ThermoFisher Scientific (Rockford, IL, USA).

### 2.2. Preparation of Dietary Antigens

Food antigens were prepared from products purchased from the supermarket in either raw, roasted, or cooked form. Cooked food proteins have different structural epitopes, and dietary protein preparation was conducted to reflect dietary proteins in either cooked or raw form as represented in typical human diets. For that preparation, 10 g of food product was put in a food processor using 0.1 M of phosphate buffer saline (PBS) at pH 7.4. The mixer was turned on and off for 1 hour and then kept on the stirrer overnight at 4°C. The food processor was decontaminated after each food product. After centrifugation at 20,000*g* for 15 minutes, the top layer, which contained oil bodies, was discarded. The liquid phase was removed and dialyzed against 0.01 M of PBS using dialysis bags, with a cutoff of 6000 kDa. Dialysis was repeated three times to ensure all small molecules were removed. After dialysis, protein concentrations were measured using a kit provided by Bio-Rad (Hercules, CA, USA).

### 2.3. Preparation of Dietary Oleosin Antigens

To purify the oleosin from peanuts, corn, safflower, sunflower, and soybean, the foods were prepared according to the method described above. A total of 100 mL of chloroform/methanol (2/1, *v*/*v*) was then added and blended for 2 minutes using a food processor. The mixture was put in a 50 mL tube and centrifuged at 14,000*g* for 5 minutes. The liquid in the upper phase was filtered through two layers of filter paper. The resultant filtrate was collected in multiple glass bottles and dried under a stream of air, with strong continuous agitation. The chloroform/methanol extraction step was repeated twice. A total of 20 mL of diethyl ether was then added, and the white, solid material stuck on the surface of the glass bottles was detached and resuspended in diethyl ether. At this point, 10 mL of water was added to each bottle, which was centrifuged at 20,000*g* for 5 minutes. The upper diethyl ether layer that contained lipids was removed, and the white, solid, interface material containing the oleosins was collected and transferred to microtubes with a minimum volume of water and diethyl ether. The microtubes were centrifuged at 20,000*g* for 5 minutes. The interfacial material was exposed to a stream of nitrogen to evaporate the remaining diethyl ether. One milliliter of chloroform/ethanol (95/5, *v*/*v*) was added to the interfacial material in each tube. The contents of each tube were quickly vortexed and transferred to a glass flask. To separate any protein contaminants from the oleosins, 10 mL of chloroform/methanol (95/5, *v*/*v*) was added, and the mixture was filtered through filter paper that was previously rinsed with chloroform/methanol. The filtrate was collected in a flask and dried under a stream of nitrogen. The dried oleosins were dissolved in chloroform/methanol and applied to a Sephadex LH-60 column (Bio-Rad, Hercules, CA, USA) using chloroform/methanol as the solvent. The collected fractions of oleosins were checked by sodium dodecyl sulfate- (SDS-) gel electrophoresis.

### 2.4. Preparation of Dietary Gum Antigens

Mastic gum, carrageenan, xanthan gum, guar gum, gum tragacanth, locust bean gum, and *β*-glucan were purchased from Sigma Aldrich (Saint Louis, MO, USA). Ten grams of each gum were extracted in 500 mL of buffer pH 4.6 by mixing them for 8 hours at 25°C on a magnetic stirrer. The solution was centrifuged at 20,000*g*, and supernatant was removed and concentrated by a factor of 10 using an Amicon filter. The protein concentration was measured using a kit provided by Bio-Rad (Hercules, CA, USA). All extracts were aliquoted and stored frozen at −20°C until used.

### 2.5. Enzyme-Linked Immunosorbent Assay (ELISA) for Demonstration of Immune Reactivity

Antigens and peptides were dissolved in PBS or methanol at a concentration of 1.0 mg/mL, then diluted 1 : 100 in 0.1 M carbonate-bicarbonate buffer (pH of 9.5). One hundred microliters was added to each well of the polystyrene flat-bottom ELISA plate. Plates were incubated overnight at 4 degrees Celsius and then washed three times with 200 *μ*L Tris-buffered saline (TBS) containing 0.05% Tween 20 at a pH of 7.4. The nonspecific binding of immunoglobulins was prevented by adding a mixture of 1.5% bovine serum albumin (BSA) and 1.5% gelatin in TBS, and then incubated overnight at 4°C. Plates were washed as described above, and then monoclonal or polyclonal antibodies that had been diluted 1 : 500 in 0.1 M PBS Tween containing 2% BSA were added to duplicate wells and incubated for 1 hour at room temperature.

Plates were washed, and then enzyme-labeled anti-mouse or anti-rabbit IgG antibodies were added to each well; plates were incubated for an additional 1 hour at room temperature. They were then washed five times with TBS-Tween buffer. The enzyme reaction was started by adding 100 *μ*L of paranitrophenylphosphate (PNPP) in 0.1 mL diethanolamine buffer 1 mg/mL containing 1 mM MgCl_2_ and sodium azide at a pH of 9.8. The reaction was stopped 45 minutes later with 50 *μ*L of 1N NaOH and the samples were ready for quantitative analysis by an ELISA reader. The OD was recorded at 405 nm by the microtiter reader to provide quantitative antibody reactivity levels and compared with control wells.

## 3. Results

### 3.1. Determination of Immune Reactivity Scale

Two hundred and four proteins were tested for seven target tissue antibodies in duplicate, leading to 2856 antigen-antibody OD measurements. The results of each duplicate OD were averaged together for one OD value. Of the 2856 OD measurements, the mean OD measurement was 0.25 with a standard deviation (SD) of 0.14. The OD of 0.53 represented two SD from the mean, the OD of 0.67 represented three SD from the mean, and an OD of 0.81 represented four SD from the mean. OD values below two SD or less than 0.52 were labeled nonsignificant. OD values above two SD but below three SD at OD values of 0.53–0.66 were categorized as 1+ reaction. OD values above three SD but less than four SD at OD values of 0.67–0.90 were categorized as 2+ reactions. OD values of 0.91–1.50 were categorized as 3+ reactions. OD values of 1.51–2.0 were categorized as 4+ reactions, and OD values >2.0 were categorized as 5+ reactions. The ODs of control wells coated only with BSA to which all other reagents were added were less than 0.15.

### 3.2. Immune Reactivity between Monoclonal Anti-Insulin and Anti-Insulin Receptor

#### 3.2.1. Insulin

Our investigation found no specific immune reactivity with anti-insulin antibody and any of the 204 food antigens.

#### 3.2.2. Insulin Receptors

**Figure 1:**
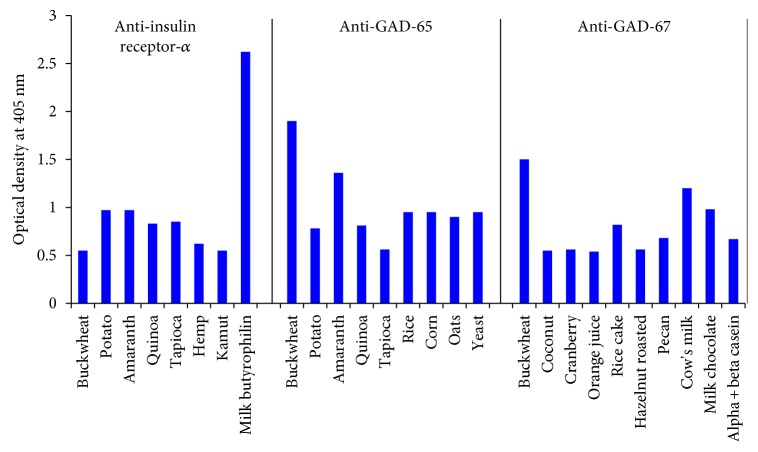
Reaction of polyclonal IR-A, GAD-65, and GAD-67 antibodies with different dietary proteins.

Our investigation identified eight dietary proteins that exhibited immune reactivity with specific IR-A antibody: milk butyrophilin (5+), potato (3+), amaranth (3+), quinoa (2+), tapioca (2+), buckwheat (1+), hemp (1+), and kamut (1+) (see Figure 1). Our investigation identified no specific targeted immune reactivity by IR-B with any of the 204 dietary proteins (see Figure 1).

### 3.3. Immune Reactivity between Polyclonal GAD-65 and GAD-67 Antibodies and Dietary Proteins

Our investigation identified 9 dietary proteins that directly exhibited immune reactivity with specific targeted GAD-65 polyclonal antibody: of these, buckwheat (3+) was the most reactive, followed by amaranth (3+), rice (3+), corn (3+), and yeast (3+), potato (2+), quinoa (2+), and oats (2+), then tapioca (1+) (see Figure 1).

### 3.4. Immune Reactivity between Monoclonal IA2 Antibodies and Dietary Proteins

**Figure 2:**
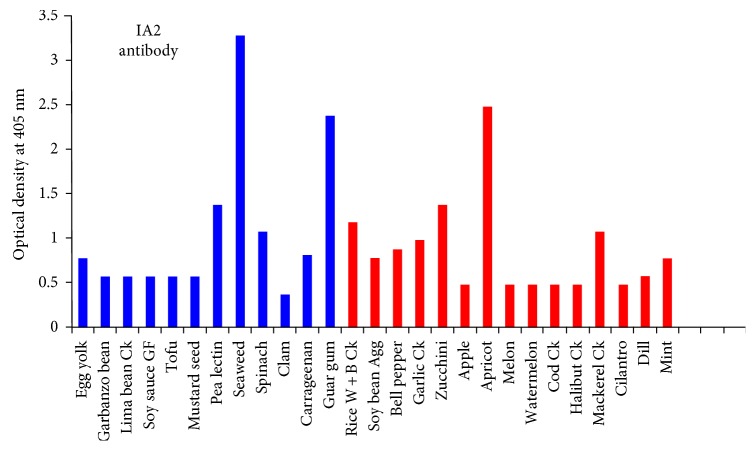
Reaction of monoclonal IA2 antibodies with different dietary proteins. The antigens in blue also reacted with ZnT8 antibodies.

Our investigation found 27 dietary proteins that exhibited immune reactivity with specific target IA2 monoclonal antibody. Of these immune reactive proteins, seaweed (5+), guar gum (5+), and apricot (5+) were the most reactive, followed by pea lectin (3+), spinach (3+), cooked white and brown rice (3+), cooked garlic (3+), zucchini (3+), and mackerel (3+), egg yolk (2+), garbanzo bean (2+), carrageenan (2+), soy bean agglutinin (2+), bell pepper (2+), and mint (2+), then cooked lima bean (1+), gluten-free soy sauce (1+), tofu (1+), and mustard seed (1+). Rice apple, melon, watermelon, clam, cooked cod, cooked halibut, and cilantro had reactions that were insignificant (see Figure 2).

### 3.5. Immune Reactivity between Monoclonal ZnT8 Antibodies and Dietary Proteins

**Figure 3:**
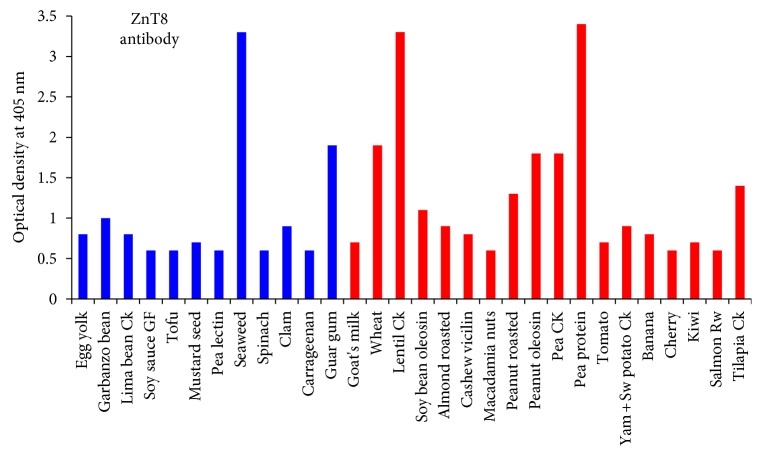
Reaction of monoclonal ZnT8 antibodies with different dietary proteins. The antigens in blue also reacted with IA2 antibodies.

The ZnT8 antibody reacted with 30 dietary proteins. The most reactive were seaweed (5+), cooked lentil (5+), and pea protein (5+), followed by guar gum (4+), wheat (4+), peanut oleosin (4+), and cooked pea (4+), garbanzo bean (3+), soy bean oleosin (3+), roasted peanut (3+), and cooked tilapia (3+), egg yolk (2+), cooked lima bean (2+), mustard seed (2+), clam (2+), goat's milk (2+), roasted almond (2+), cashew vicilin (2+), tomato (2+), cooked yam and sweet potato (2+), banana (2+), and kiwi (2+), then gluten-free soy sauce (1+), tofu (1+), pea lectin (1+), spinach (1+), carrageenan (1+), macadamia nut (1+), cherry (1+), and salmon (1+) (see Figure 3).

In addition, 10 dietary proteins that directly exhibited immune reactivity with specific targeted GAD-67 polyclonal antibody: buckwheat (4+) was the most reactive, followed by cow's milk (3+); milk chocolate (3+) were the most reactive, followed by raw and roasted pecan (2+), alpha and beta casein (2+), and rice cake (2+), then coconut (1+), cranberry (1+), orange juice 1+), and roasted hazelnut (1+) (see Figure 1).

## 4. Discussion

There is some evidence showing that pancreatic beta islet cell antibodies in children are predictive for determining progression to diabetes using proportional hazards analysis [34]. Early identification of potential immunological reactive dietary protein triggers may aid clinicians in devising stratagems and protocols to reduce inflammatory sequelae and progression of the disease process in susceptible subgroups [35].

The focus of our laboratory research was to identify food proteins that have the potential to cross-react with pancreatic islet cells by evaluating immune reactivity between antibodies made against target tissue antigens involved in diabetes and various dietary proteins. The foods found to be immune reactive to pancreas target sites included the main food groups of gluten proteins, nongluten grains, and dairy proteins. Food groups tend to have homologous amino acid sequences. Reactivity to one food may also lead to immune reactivity with other dietary proteins in that food group. For example, several papers demonstrate that an isolated allergy to a single fish species leads to immune reactivity to other fish species due to amino acid structural similarity [36–39]. Coallergy reactions with legumes that share similar structural similarity were also identified by Bernhisel-Broadbent et al. [36], where they found that 37% of children immune reactive to one legume had reactions against all six major legumes, and 79% had binding to at least another legume [40]. Immune sensitivity with natural rubber latex allergens have also been reported to lead to cross-reactive sensitivity to foods that contain prohevein, such as banana, avocado, kiwi, chestnut, potato, and papaya, in what is called “latex-fruit syndrome” [41, 42].

In this current study, we examined possible cross-reactivity between islet cell antigens and different food proteins by measuring the reactivity of highly specific antibodies made against islet cell antigens with a variety of food antigens.

With IR-A, the strongest reaction was with milk butyrophilin, while with GAD-65, the strongest reactions were with buckwheat, amaranth, rice, corn, and yeast. With GAD-67, the strongest reactions were with buckwheat, cow's milk, and milk chocolate (see Figure 1).

With IA2 antibody, the strongest reactions were with seaweed, guar gum, and apricot, then pea lectin, spinach, cooked white, and brown rice, cooked garlic, zucchini, and mackerel (see Figure 2).

With ZnT8 antibody, the strongest reactions were with seaweed, cooked lentil, and pea protein, followed by guar gum, wheat, peanut oleosin, and cooked pea, then garbanzo bean, soy bean oleosin, roasted peanut, and cooked tilapia (see Figure 3).

Overall, we found that IA2 antibody reacted with 27 food proteins, ZnT8 antibody reacted with 30 food proteins, and GAD-65 reacted with 9 food proteins. Out of these foods, the same 12 foods reacted with both IA2 and ZnT8 antibodies. These 12 foods were egg yolk, garbanzo bean, cooked lima bean, gluten-free soy sauce, tofu, mustard seed, pea lectin, seaweed, spinach, clam, carrageenan, and guar gum. The detection of these dietary proteins cross-reactive with these major islet cell antigens, IA2, ZnT8, and GAD-65, especially the 12 foods that are dually reactive to both IA2 and ZnT8, warrants further investigation, because on the occasion of oral tolerance failure, if these foods manage to penetrate the epithelial barriers, the production of cross-reactive antibodies may contribute towards the destruction of islet cells.

Both IA2 and ZnT8 were also shown to be reactive to pea lectin (3+ and 1+, resp.). Lectins stimulate class II human leukocyte antigens (HLAs) of islet cells, which normally do not display them. Islet cells carry a very specific disaccharide determinant called N-acetyllactosamine, to which wheat, peanuts, soy, potato, and tomato lectins love to bind. This binding can result in islet cells expressing the class II HLAs and foreign antigens together, making the individual susceptible to autoimmune attack.

The ZnT8 antibody reacted not only with roasted peanut (3+) but also with peanut oleosin (4+) and soy bean oleosin (3+). Oleosins are relatively small (15 to 25 kDa) oil proteins that provide energy for plant seed cells. To a sensitive person, the ingestion of even a small amount can provoke severe reactions. Refined vegetable oils are used in a wide variety of food products, but oils from plants such as peanuts and soybeans have been recognized as potent antigens and allergens [43, 44].

Both IA2 and ZnT8 reacted very strongly to guar gum (5+ and 4+, resp.). Both also reacted to a lesser extent to carrageenan (2+ and 1+, resp.). Like oil, gums have an extremely broad range of commercial and industrial use, in not just food but including pharmaceuticals, printing, and other applications. Even though gums are generally recognized as safe by the FDA, gums do have a history of association with sensitivities or allergic reactions [45]. The fact that both of our tested islet cell antigens reacted strongly with gums raises concerns about the wide variety of commonly available products containing gums that may contribute to islet cell autoimmunity through the mechanism of this demonstrated cross-reactivity.

Aquaporin 4 (AQP4) is a class of water channels found in many cells of the body including the stomach, brain, lung, and skeletal muscle. Aquaporin is also found in many plants, and a recent study showed a significant similarity between the amino acid sequences of soy, spinach, corn, tomato, and tobacco with human aquaporin epitope 207–232 to elicit concerns about cross-reactivity. IA2 and ZnT8 had reactions in varying degrees with spinach, tomato, and different soy antigens (see Figure 2) [46].

Wheat has long been confirmed to be associated with a broad spectrum of autoimmune disorders, including T1D [44]. ZnT8 reacted strongly with wheat (4+). Considering the degree of cross-reactivity between wheat/gliadin/gluten and different neuronal antigens such as GAD-65, it should not be surprising that so many autoimmune reactivities can arise, and the significance of this reaction with wheat should be looked at closely. Animal models of autoimmune diabetes found that 6 months of a gluten-free diet had a beneficial effect on the preservation of islet cell function [47]. A population-based screening study of Danish children with autoimmune diabetes and celiac disease found improvements with a gluten-free diet in height and weight development during a 2-year follow-up [48].

The role of milk proteins and their involvement with autoimmune diabetes due to mimicry epitopes has previously been reported from epidemiological studies and from animal models that found that milk proteins play a diabetogenic role [21]. Our study showed that the islet cell antigens reacted with goat's milk, milk butyrophilin, cow's milk, milk chocolate, and *α*+*β* casein. Exposure to cow's milk proteins may prime the immune system to recognize and react to islet cell antigens that possess sequence homology to milk proteins, as reviewed by Vojdani [45].

Although many researchers have previously investigated the potential role of gluten and autoimmune diabetes, our largest immune reactive category was to nongluten grains, which has been less studied. These foods accounted for 4.4% of the screened dietary proteins and included rice, quinoa, hemp, yeast, oats, buckwheat, amaranth, and tapioca. This nongluten grain category was highly reactive to GAD-65 and insulin receptor alpha (see Figure 1, Table 2). The potential immune reactive role of gluten-free grains in susceptible subjects—such as celiac disease patients with autoimmune diabetes—is of concern, as those grains are often used as a substitute grain for individuals on a gluten-free diet. Many of these gluten-free grains demonstrated strong 3+ and even 4+ immunological reactions. Vojdani et al. previously published how these gluten-free grain proteins can also react with *α*-gliadin 33-mer antibody and explained why some individuals with gluten sensitivity do not respond to a gluten-free diet alone [49]. The results of our investigation show that many gluten-free grains that cross-react with gluten also have immune reactivity to pancreatic target sites. Immune reactivity to these grains may play a role in diabetes progression independent of gluten exposure or the high glycemic index of grain products.

The second largest reactive food category we identified was bean proteins. Beans (including black, white, navy, lima, pinto, garbanzo, soy, and kidney) are a winning combination of high-quality carbohydrates, lean protein, and soluble fiber that helps stabilize your body's blood-sugar levels and keeps hunger in check. Unfortunately, although they are generally considered a low glycemic index food, in our study, the islet cell antigens reacted with garbanzo bean, lima bean, gluten-free soy sauce, soy bean oleosin, soy bean agglutinin, tofu, pea lectin, pea protein, cooked pea, and cooked lentil.

Our investigation identified 49 dietary proteins (24% of the 204 proteins) that are classified as low glycemic foods (glycemic index <55) to be reactive to antibodies made against pancreatic autoimmune target sites (see Table 3). Foods such as high-fiber grains, nuts, and dairy products that are encouraged for both type 1 and 2 diabetes patients were found to be highly reactive to antibodies against insulin receptors, islet cells, GAD-65, and GAD-67 (see Figures 1() and 2(), Table 2). Current diabetes dietary management encourages the consumption of these foods [50]. Because the etiology of both juvenile type 1 diabetes and LADA is immunological, we suggest that future dietary management should include not only glycemic management but also the reduction or avoidance of immunoreactive dietary antigens shown to have a role in the etiology of autoimmune diabetes.

We must not forget that immune or sensitive reactions to food can also depend on whether the food is raw or cooked. A food in its natural state can be vastly different from the same food that has been cooked, processed, or modified. The food can react with sugar, lipids can be oxidized, peptide chains can be broken, and neoantigens can be formed. Thus, an individual that can eat a raw food safely may be highly reactive to the same food if cooked, or vice versa. It is not surprising then that in our tests, IA2 reacted strongly with cooked garlic (3+) but less with raw garlic (insignificant), strongly with pea lectin (4+) but not as much with pea protein (insignificant), and moderately with apple (2+) but less with apple cider (1+). In a similar fashion, ZnT8 reacted moderately with raw banana (2+) but less with cooked banana (insignificant), stronger against raw salmon (3+) but not as much with cooked salmon (1+), and very strongly with pea protein (5+) but less with pea lectin (3+).

It is important to note that cross-reactivity alone is not likely to initiate the pathogenesis of autoimmunity. However, the potential exists to upregulate subclinical or preexisting autoimmune conditions, and we suggest that immune reactivity is likely to promote the autoreactive process in susceptible subgroups if exposed to the specific antigens [51]. This susceptibility to dietary protein immune reactivity may occur if the gut immune system loses oral tolerance homeostasis and mucosal immune function, as found in autoimmunity [52, 53]. A cause for concern is that mechanisms of intestinal permeability, chronic intestinal inflammation, and impaired mucosal immune regulatory mechanisms have all been reported in children with type 1 diabetes [54].

These underlying mechanisms that may coexist with autoimmune diabetes and, in combination with immune reactive foods that share amino acid sequence homology, may lead to dietary proteins that can be diabetogenic in a subset of individuals. Further research to investigate the pathogenic role of these dietary proteins is necessary but must include confounding variables such as humoral and cellular immunity factors, intestinal permeability, mucosal regulatory mechanisms, and other factors that can determine whether exposure to dietary proteins is antigenic or immunologically benign; protein sequence similarity alone would not account for a pathogenic role. It is unlikely that the consumption of these dietary proteins alone would be diabetogenic in nonsusceptible populations. However, the immunoreactive dietary proteins we identified may act as cross-reactive food antigens for subsets of individuals who produce these antibodies due to loss of immunological tolerance.

## 5. Conclusion

The results of our study identified immune reactivity between antibodies to insulin, insulin receptors, islet cell antigens, and food antigens. Potential tissue antibody binding with various food antigens or food antibody binding to specific pancreatic sites can lead to the possibilities that some dietary proteins may play an antigenic role with autoimmune diabetes.

Even though many of the individual proteins in these groups may be considered safe or of low glycemic index, the consumption of these foods by a sensitive or predisposed individual may trigger immune reactions or autoimmunities.

Further research should be conducted to evaluate the specific epitope for each of the dietary proteins that react with these tissue-specific antibodies. It is necessary to determine the exact amino acid sequence homology, the immunological factors that convert dietary proteins from benign to pathogenic, and the clinical role that these dietary proteins play in autoimmune diabetes. However, the results of our research provide a first step in narrowing down a list of specific dietary proteins that, due to cross-reactivity, may potentially have an impact on autoimmune diabetes.

## Figures and Tables

**Table 1:** Dietary proteins screened for immune reaction.

*Dairy and eggs, modified*	Flax seed	Seaweed	Imitation crab
Alpha-casein & beta-casein	Hazelnut, raw + roasted	Spinach + Aquaporin	Clam, boiled
Cow's milk	Macadamia nut, raw + roasted	Tomato + Aquaporin	Oyster, boiled
Chocolate milk	Mustard seed	Tomato paste	Scallops, seared
Egg white, boiled	Pecan, raw + roasted	Yam + Sweet potato, baked	Squid (Calamari), seared
Egg yolk, boiled	Peanut, roasted	Zucchini, boiled	Shrimp, seared
Goat's milk	Peanut butter	*Fruit, raw and modified*	Shrimp tropomyosin
Milk butyrophilin	Peanut agglutinin	Apple	Parvalbumin
Soft cheese + Hard cheese	Peanut oleosin	Apple cider	*Meat, modified*
Whey protein	Pistachio, raw + roasted	Apricot	Beef, boiled medium
Yogurt	Pumpkin seeds, roasted	Avocado	Chicken, boiled
*Grains, raw and modified*	Sesame albumin	Banana	Lamb, baked
Amaranth	Sesame oleosin	Banana, boiled	Pork, baked
Buckwheat	Sunflower seeds, roasted	Latex hevein	Turkey, baked
Casomorphin	Walnut	Blueberry	Gelatin
Oats	*Vegetables, raw and modified*	Cantaloupe + Honeydew melon	Meat glue
Quinoa	Artichoke, boiled	Cherry	*Herbs, raw*
Rice	Asparagus	Coconut, meat + milk	Basil
Rice, white + brown, boiled	Asparagus, boiled	Cranberry	Cilantro
Rice cake	Beet, boiled	Date	Cumin
Rice protein	Bell pepper	Fig	Dill
Rice endochitinase	Broccoli	Grape, red + green	Ginger
Rye, barley, spelt, polish wheat	Broccoli, boiled	Red wine	Oregano
Sesame	Brussels Sprouts, boiled	White wine	Parsley
Sorghum	Cabbage, red + green	Grapefruit	Rosemary
Tapioca	Cabbage, boiled	Kiwi	Thyme
Teff	Canola oleosin	Lemon + Lime	*Spices, raw*
Wild rice, boiled	Carrot	Mango	Cinnamon
Wheat + Alpha-gliadins	Carrot, boiled	Orange	Clove
Yeast miller	Cauliflower, boiled	Orange juice	Mint
Hemp	Celery	Papaya	Nutmeg
*Beans, modified*	Chili pepper	Peach + Nectarine	Paprika
Black bean, boiled	Corn + Aquaporin, boiled	Pear	Turmeric (Curcumin)
Bean agglutinins	Popped corn	Pineapple	Vanilla
Dark chocolate + Cocoa	Corn oleosin	Pineapple bromelain	*Gums*
Fava bean, boiled	Cucumber, pickled	Plum	Carrageenan
Garbanzo bean, boiled	Eggplant, boiled	Pomegranate	Gum guar
Kidney bean, boiled	Garlic	Strawberry	Gum tragacanth
Lentil, boiled	Garlic, boiled	Watermelon	Locust bean gum
Lentil lectin	Green Bean, boiled	*Fish and seafood, raw and modified*	Mastic gum + Gum arabic
Lima bean, boiled	Lettuce	Cod, baked	Xanthan gum
Pinto bean, boiled	Mushroom, raw + boiled	Halibut, baked	*Brewed beverages and additives*
Soybean agglutinin	Okra, boiled	Mackerel, baked	Coffee bean protein, brewed
Soybean oleosin + Aquaporin	Olive, green + black, pickled	Red Snapper, baked	Instant coffee
Soy Sauce, gluten-free	Onion + Scallion	Salmon	Black tea, brewed
Tofu	Onion + Scallion, boiled	Salmon, baked	Green tea, brewed
*Nuts and seeds, raw and modified*	Pea, boiled	Sardine + Anchovy, cooked	Honey, raw + processed
Almond	Pea protein	Sea bass, seared	Beta-glucan
Almond, roasted	Pea lectin	Tilapia, baked	Food coloring
Brazil nut, raw + roasted	Potato, white, baked	Trout, baked	
Cashew	Potato, white, fried	Tuna	
Cashew, roasted	Pumpkin + Squash, boiled	Tuna, seared	
Cashew vicilin	Radish	Whitefish, baked	
Chia seed	Safflower + Sunflower oleosin	Crab + Lobster, boiled	

**Table 2:** Reactivity of antibodies against pancreatic islet cell antigens with food proteins.

Food protein	Reactivity
*Gluten*	
Wheat	ZnT8 (4+)
Kamut	IR-A (1+)

*Nongluten Grains*	
Buckwheat	GAD-65 (4+), GAD-67 (3+), IR-A (1+)
Amaranth	GAD-65 (3+), IR-A (3+)
Quinoa	GAD-65 (2+), IR-A (2+)
Tapioca	IR-A (2+), GAD-65 (1+)
Oats	GAD-65 (2+)
Rice/rice cake	GAD-65 (3+), IA2 (3+), GAD-67 (2+)
Hemp	IR-A (1+)
Corn	GAD-65 (3+)

*Beans & legumes*	
Lentil	ZnT8 (5+)
Pea protein	ZnT8 (5+)
Pea cooked	ZnT8 (4+)
Pea lectin	IA2 (3+)
Garbanzo bean	ZnT8 (3+), IA2 (2+)
Lima bean	ZnT8 (2+), IA2 (1+)
Soy sauce gluten free	ZnT8 (1+), IA2 (1+)
Tofu	ZnT8 (1+), IA2 (1+)
Soy bean agglutinin	IA2 (2+)
Soy bean oleosin	ZnT8 (3+)

*Seafood*	
Clam	ZnT8 (2+)
Mackerel	IA2 (3+)
Salmon	ZnT8 (1+)
Tilapia	ZnT8 (3+)

*Others*	
Seaweed	ZnT8 (5+), IA2 (5+)
Egg yolk	ZnT8 (2+), IA2 (2+)
Yeast	GAD-65 (3+)
Mustard seed	ZnT8 (3+), IA2 (1+)

*Milk proteins*	
Goat's milk	ZnT8 (2+)
Cow's milk	GAD-67 (3+)
Milk chocolate	GAD-67 (3+)
Milk butyrophilin	IR-A (5+)
*α*+*β* casein	GAD-67 (2+)

*Nuts*	
Hazelnut	GAD-67 (1+)
Pecan	GAD-67 (2+)
Almond roasted	ZnT8 (2+)
Cashew vicilin	ZnT8 (2+)
Macadamia	ZnT8 (1+)
Peanut roasted	ZnT8 (3+)
Peanut oleosin	ZnT8 (4+)

*Gums*	
Guar gum	IA2 (5+), ZnT8 (4+)
Carrageenan	IA2 (2+), ZnT8 (1+)

*Fruit*	
Apricot	IA2 (5+)
Banana	ZnT8 (2+)
Cherry	ZnT8 (1+)
Kiwi	ZnT8 (2+)
Coconut	GAD-67 (1+)
Cranberry	GAD-67 (1+)
Orange juice	GAD-67 (1+)

*Vegetables*	
Bell pepper	IA2 (2+)
Garlic Ck	IA2 (2+)
Tomato	ZnT8 (2+)
Potato	IR-A (3+), GAD-65 (2+)
Yam + Sw Potato	ZnT8 (2+)
Zucchini	IA2 (3+)

*Herbs*	
Dill	IA2 (1+)
Mint	IA2 (2+)

**Table 3:** Low glycemic index foods reactive with pancreatic islet cell antibodies.

Milk butyrophilin	Nuts	Corn
Cow's milk	Oats	Coconut
*α*+*β* casein	Quinoa	Cranberry
Goat's milk	Rice	Hazelnut
Milk chocolate	Hemp	Pecan
Guar gum	Seaweed	Vegetables
Carrageenan	Beans & Legumes	Seafood

Glycemic index <55.
